# Prenyl Ammonium Salts – New Carriers for Gene Delivery: A B16-F10 Mouse Melanoma Model

**DOI:** 10.1371/journal.pone.0153633

**Published:** 2016-04-18

**Authors:** Emilia Grecka, Malgorzata Statkiewicz, Agnieszka Gorska, Marzena Biernacka, Monika Anna Grygorowicz, Marek Masnyk, Marek Chmielewski, Katarzyna Gawarecka, Tadeusz Chojnacki, Ewa Swiezewska, Maciej Malecki

**Affiliations:** 1 Department of Molecular and Translational Oncology, Maria Sklodowska-Curie Memorial Cancer Center and Institute of Oncology, Warsaw, Poland; 2 Department of Pharmacology, National Research Institute of Mother and Child, Warsaw, Poland; 3 Department of Genetics, Maria Sklodowska-Curie Memorial Cancer Center and Institute of Oncology, Warsaw, Poland; 4 Department of Applied Pharmacy and Bioengineering, Medical University of Warsaw, Warsaw, Poland; 5 Department of Immunology, Maria Sklodowska-Curie Memorial Cancer Center and Institute of Oncology, Warsaw, Poland; 6 Institute of Organic Chemistry PAS, Warsaw, Poland; 7 Institute of Biochemistry and Biophysics PAS, Warsaw, Poland; University of Quebec at Trois-Rivieres, CANADA

## Abstract

**Purpose:**

Prenyl ammonium iodides (Amino-Prenols, APs), semi-synthetic polyprenol derivatives were studied as prospective novel gene transfer agents.

**Methods:**

AP-7, -8, -11 and -15 (aminoprenols composed of 7, 8, 11 or 15 isoprene units, respectively) were examined for their capacity to form complexes with pDNA, for cytotoxicity and ability to transfect genes to cells.

**Results:**

All the carriers were able to complex DNA. The highest, comparable to commercial reagents, transfection efficiency was observed for AP-15. Simultaneously, AP-15 exhibited the lowest negative impact on cell viability and proliferation—considerably lower than that of commercial agents. AP-15/DOPE complexes were also efficient to introduce pDNA to cells, without much effect on cell viability. Transfection with AP-15/DOPE complexes influenced the expression of a very few among 44 tested genes involved in cellular lipid metabolism. Furthermore, complexes containing AP-15 and therapeutic plasmid, encoding the TIMP metallopeptidase inhibitor 2 (TIMP2), introduced the *TIMP2* gene with high efficiency to B16-F10 melanoma cells but not to B16-F10 melanoma tumors in C57BL/6 mice, as confirmed by TIMP2 protein level determination.

**Conclusion:**

Obtained results indicate that APs have a potential as non-viral vectors for cell transfection.

## Introduction

Gene therapy, which implies genetic material to be introduced to the target cells as a therapeutic substance, is nowadays considered as a promising alternative approach to pharmaceutical treatment of many diseases. So far this approach has been successful in a limited number of clinical applications, e.g. adeno-associated virus (AAV1) delivery of a transgene encoding lipoprotein lipase (LPL) to patients suffering from LPL-deficiency (LPLD) [1]. A key element, determining the success of this strategy is an effective and safe introduction of the therapeutic nucleic acid into the cells. Therefore intensive research on development of carriers of genetic material, which can address the challenges of gene delivery, is constantly conducted. In the clinical trials performed so far viral vectors seem the most commonly used as very effective gene delivery vehicles [2]. However, numerous concerns are raised related to the safety of their employment in patient treatment—triggering of acute inflammatory response as well as delayed humoral and cellular immune response, risk of insertional mutagenesis, etc. These obstacles have led to the exploration of alternative methods of transfection [3]. Nowadays, an increasing number of clinical protocols describe applications of nonviral gene therapy formulations, which in comparison to viral vectors, are less immunogenic, capable of introducing genes of unlimited size, and also cheap to produce on a large scale [4].

Cationic carriers (lipids or polymers) are among various compounds studied up to date which, due to the presence of positively charged groups, are thought to interact electrostatically with the negatively charged phosphate groups of pDNA and form carrier:pDNA complexes (with simultaneous pDNA packing and condensation) [5]. Transfection effectiveness of cationic lipids depends, among others, on their structure, e.g. a geometric shape of the molecule, the number of charged groups per lipid molecule and hydrophobic properties of the lipid moiety. Moreover, several parameters characterizing the formula used for transfection are also of importance, e.g. the ratio of the positive charge of cationic lipid to the negative charge of pDNA of interacting molecules, the size of the thus formed complex, and the effect of eventually used helper lipid(s) (also called co-lipids) [3]. Results of numerous studies indicate that the addition of a helper lipid (for example dioleoylphosphatidylethanolamine, DOPE) to cationic lipids can enhance the stability of lipoplexes under physiological conditions and also increase the transfection efficiency [6]. Various cationic lipids and polymers were employed in such applications and a few examples of the successful application of cationic lipids as components of transfection mixtures are presented below. Giron-Gonzalez et al. [7] showed that complexes containing polyethyleneimine (PEI) and citric acid (CA) transfected the several cell lines with higher efficiency than Lipofectamine or PEI and also promoted *in vivo* transfection in mouse with an enhanced efficiency compared to PEI. Zakharova et al. [8] tested hydroxyethylated gemini surfactants with varied spacer length/pGFP (plasmid DNA encoding for green fluorescent protein) complexes and showed that transfection of eukaryotic cells with use of some of the formulations resulted in considerably high transfection efficiency (about 50–75% of GFP-positive cells). Parvizi et al. [9] showed that lipoplex formulations based on cationic pyridinium-based lipids co-formulated with the cationic lipid 1,2-dimyristoyl-sn-glycero-3-ethylphosphocholine (EPC) and cholesterol transfected cells with efficiency similar to obtained with use of Lipofectamine2000.

However, lower transfection efficacy than that of viral vectors, especially *in vivo*, observed for so far explored cationic lipids on the one hand, and their substantial toxicity towards the cells on the other, stimulate continuous search for novel carriers.

**Fig 1:**
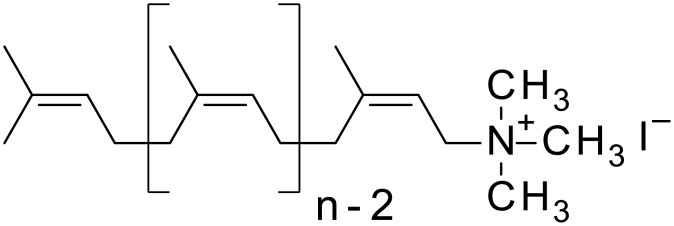
Structure of Amino-Prenols, the AP molecule contains an polyisoprenoid chain composed of n isoprene residues.

Polyprenyltrimethylammonium iodides, further called Amino-Prenols (APs) are cationic, semi-synthetic derivatives of polyprenols (Fig 1) [10]. Polyprenols are common constituents of plant photosynthetic tissues (for review see [11]). Together with dolichols, typically found in mammalian tissues, they constitute a group of polyisoprenoid lipids. Besides their role as cofactors in protein glycosylation and prenylation [12] polyisoprenoid alcohols are postulated to act as membrane fluidizers, fusiogenic agents [13] and components of the intracellular protein-transporting machinery [14]. Taking into account their fusogenic activity a concept of the feasibility of cationic derivatives of polyprenol as components of lipofecting formula was proposed and appropriate studies have been initiated. As a result good efficacy of *in vitro* transfection with application of AP-7-based (aminoprenol composed of seven isoprene units) complexes has been demonstrated [10]. Further studies on AP-7 as a drug carrier in rats revealed liposomes containing AP-7 to be harmless to the experimental animals [15]. AP-7 containing liposomes did not affect functions of the excretory and cardiovascular systems, nor renal morphology [16]. These encouraging results are a good starting point for developing new, safe and efficient formulas of gene delivery.

TIMP2 is a member of the family of tissue inhibitors of metallopeptidases (TIMPs).The results of *in vitro* and *in vivo* studies showed that TIMP2 plays an important role in tumor progression. It was demonstrated that overexpression of TIMP2 in melanoma cells and cells of other types of cancer inhibits the tumor growth, angiogenesis, tumor invasiveness and metastasis [17–19]. Therefore we also studied the efficiency of APs to deliver *TIMP2* gene to mouse melanoma cell line B16-F10 and to B16-F10 tumors in mice.

In the present study we tested the transfectional properties of polyprenol-based cationic lipids: AP-7, AP-8, AP-11 and AP-15 on a mouse melanoma cell line (B16-F10). The transfection efficiency was evaluated for two types of formulations: complexes AP:pDNA and complexes AP/DOPE:pDNA. The carrier:pDNA ratio was optimized. The viability of transfected cells was studied utilizing the crystal violet test. The expression of selected genes related to lipid metabolism in transfected cells was analyzed using the qPCR method. The efficiency of gene delivery after intratumoral administration to mice bearing B16-F10 melanoma tumors was evaluated by means of the PCR technique, whereas the expression of the introduced gene was assessed at the protein level by means of Western blot analysis. This study demonstrates that AP-15 is an effective and safe carrier for *in vitro* gene transfer.

## Material and Methods

### Cell culture

B16-F10 mouse melanoma (ATCC^®^ CRL-6475^™^) and L1 mouse sarcoma cells derived from a lung metastasis of a BALB/c mouse [20] were cultured in Dulbecco’s Modified Eagle Medium (Gibco BRL, Life Technologies) supplemented with 10% FBS (Lonza, BioWhittaker). Cells were maintained at 37°C in a humidified 5% CO_2_ atmosphere with media changes every third day.

### Plasmids

The plasmids pAAV-LacZ—pLacZ (Stratagene), pAAV-IRES-hr-GFP—pGFP (Stratagene), pSecTag2B –pSec (Invitrogen) and pSecTag2B/TIMP2 –pTIMP2, were used. pTIMP2 was obtained by insertion of the coding sequence of *TIMP2* (NM_003255) into the pSecTag2B plasmid (Invitrogen) according to the manufacturer’s instructions.

### Polyprenol isolation and polyprenyltrimethylammonium iodide synthesis

Prenol-7, -8, -11 and -15 were from the Collection of Polyprenols, Institute of Biochemistry and Biophysics, Polish Academy of Sciences, Warsaw, Poland. Aminoprenols were prepared following the previously described method ([7]; Patent No 211824 Patent Office of the Republic of Poland, 26.06.2012) with some modifications. Briefly, Prenol-7, -8, -11 or -15 were used as substrates to prepare cationic derivatives. Pure APs were dissolved in chloroform:methanol 2:1, by vol. and stored at -20°C until used. A sample of AP solution was evaporated under a stream of nitrogen, dissolved in absolute ethanol and used to prepare two types of lipid formulas.

### Agarose gel retardation assay

Lipid:pDNA complexes were defined by the ratio of cationic lipid nitrogen to DNA phosphate (N/P) [21] whereas lipid/DOPE:pDNA complexes by the amount of lipids versus amount of pDNA (μg/μg).

AP:pGFP and PEI:pGFP (polyethylenimine, Sigma) complexes were prepared at various N/P ratios in the range 0.33–5.66. Ethanolic solution of AP (20 mg/ml) was diluted with water and mixed carefully (final concentration of AP was 1mM in ethanol:water 1:15.6, by vol.). PEI was dissolved in water (final concentration was 1mM). N/P ratios were calculated as follows: nitrogen of carrier per nucleic acid phosphate, where 1 μg of DNA corresponds to 3 nmol of phosphate and 1 μl of PEI solution or AP solution contains 1 nmol of amine nitrogen. Complexes were prepared in 0.9% NaCl by gentle vortexing of a mixture of pDNA (2 μg) and either AP (2–34 μl) or PEI (6–21 μl) solutions.

AP-15/DOPE:pGFP and AP-15/DOPE/DMEM:pGFP complexes were prepared using different amounts of lipids in the range 2.5–25 μg of lipids/μg of pDNA. AP-15 and helper lipid dioleoylphosphatidylethanolamine (DOPE, Sigma) dissolved in absolute ethanol (concentration of 10 mg/ml and 3.2 mg/ml, respectively) were mixed to obtain mixtures at 1.5:1 AP-15 to DOPE molar ratio. Where indicated the lipofecting formulations were prepared either in DMEM or in aqua pro injectione (Polpharma). The components were mixed and stored at 4°C. The transfection mixtures was extensively vortexed prior to use. The complexes were prepared in DMEM with the 2 μg of pDNA.

The complexes were incubated at room temperature for 20 min, or for 30 min in case of DOPE-containing formulations, supplemented with 10 μl of 6X DNA Loading Dye (Thermo Scientific) solution, loaded onto 0.8% (w/v) agarose gel containing EtBr (0.5 μg/mL) and separated electrophoretically (45 min at 90 V). The gels were analyzed using Multi- Analyst 1.1 (BIO-RAD) to determine pDNA location.

### Cell transfection procedures

#### Transfection using AP-based complexes

The cells were seeded at the density of 1.3–1.5 x 10^5^ cells per 3.5 cm diameter dish, or 2–3 x 10^5^ cells per 6 cm diameter dish, in DMEM (Dulbecco’s Modified Eagle Medium) with 10% of FBS and incubated for 24h (approx. 60–70% confluence). Before transfection, cell culture medium was replaced with DMEM without FBS. Complexes were prepared in 0.9% NaCl by gentle vortexing a mixture of pDNA and AP (1mM in ethanol: water 1:15.6, by vol.), or PEI aqueous solution (1 mM), and incubated for 20 min. The complexes containing AP or PEI were added to the cell culture (from 4 to 8 μg of pDNA, various carrier:pDNA (N/P) ratios were tested). The cells were incubated for 5 hours and in the case of AP-15/AP-11 the culture was subsequently supplemented with the an equal volume of 20% FBS (final concentration of FBS– 10%), while for AP-7/AP-8 and PEI the media were replaced with fresh DMEM with 10% FBS. The incubation was continued for 43h. The transfection efficiency was evaluated by flow cytometry (pGFP) or β-Gal assay (pLacZ).

Control experiments with application of nextgeneration lipid reagent Attractene Transfection Reagent (Qiagen) and cationic polymer Satisfection Transfection Reagent (Agilent Technologies) were conducted according to the manufacturer’s recommendations, using the experimentally verified most effective pDNA amounts and reagents volumes.

#### Transfection with use of AP-15/DOPE:pDNA complexes

Transfection was performed following the previously described procedure [10]. B16-F10 cells were seeded one day before transfection into 24-well plates at a density of 3.5–4.5 × 10^3^/well, or into 3.5 cm diameter dishes at the density of 1.3–1.5 x 10^5^ cells, in DMEM with 10% FBS and grown to 60–70% confluence. Before transfection, cell culture medium was removed, subsequently cells were carefully rinsed with serum-free medium and the latter medium was applied on the cells. The complexes were prepared in DMEM without FBS by gentle vortexing the mixtures containing pGFP and AP-15/DOPE or AP-15/DOPE/DMEM (AP-15: DOPE molar ratio was 1.5:1), and incubated at room temperature for 30 min. AP-15/DOPE:pGFP or AP-15/DOPE/DMEM:pGFP complexes were added to the cell culture (2 or 4 μg of pGFP, various amounts of lipids were tested) and incubation was carried out for 5 hours; the culture was subsequently supplemented with the an equal volume of 20% FBS and further incubated for 43 hours. The transfection efficiency was evaluated by fluorometry or flow cytometry.

Control experiments with Attractene Transfection Reagent (Qiagen) and Lipofectamine3000 Transfection Reagent (Life Technologies) were conducted according to the manufacturer’s recommendations.

### Flow Cytometry

48 hours post-transfection the cells were detached with 0.5 ml of 0.05% trypsin—EDTA (Lonza) and centrifuged. Supernatant was removed, and the cells were washed twice in PBS. The cells were resuspended in 100 μl of 7-AAD Staining Solution (BD Biosciences) (0.1 μg/sample) prepared in PBS. The cells were incubated on ice for 10 min and supplemented with 400 μl of PBS. The samples were analysed using a BD FACSCanto ll flow cytometer with FACSDiva 6.1.3 software (BD Biosciences). The samples were collected using high flow rate and time as stopping criterion (35 seconds). The excitation wavelength was 488 nm with emission detected with a photomultiplier equipped with a 530/30 band pass filter (GFP) and 670 long pass filter (7-AAD). Transfection efficacy was estimated by quantification of GFP-positive cells in the sample. Control samples, i.e. cells not subjected to transfection as well as cells treated with suitable amounts of respective AP, AP-15/DOPE, AP-15/DOPE/DMEM, DOPE, AP-15/DOPE:pSec or AP-15/DOPE/DMEM:pSec complexes were analyzed in order to determine the autofluorescence of cells. The population of viable cells in the sample was determined by discrimination of 7-AAD labeled dead cells. Total cell count was presented as a percentage of cells normalized to the total number of non-transfected control cells. Viable cell counts was presented as a percentage of viable cells, transfected with the complexes or treated with the respective reagents alone, normalized to the number of viable, non-treated control cells. The experiments were done with n = 3–6 replicates.

### β-galactosidase activity assay

β-gal assay was performed using protocols described earlier [22] with modifications. Cell pellets were washed twice with PBS and suspended in 50–100 μl of 0.25 M Tris-HCl pH 8.0 buffer. Three freeze-thaw cycles were performed to lyse cells. The samples were centrifuged for 10 minutes at 12 000 rpm at 4°C. 3 μl of 100 × Mg buffer (0.1 M MgCl_2_, 4.5 M β-mercaptoethanol), 50 μl of 2-nitrophenyl β-D-galactopyranoside (4 mg/mL ONPG (Sigma) in 0.1 M sodium phosphate) substrate solution were added to the protein lysates. After 30 min of incubation at 37°C the reaction was stopped by adding 500 μl of 1 M Na_2_CO_3_ and the absorbance of the supernatants was measured at 420 nm to estimate the amount of o-nitrophenol in each sample. β-galactosidase activity was presented as the ratio of the o-nitrophenol amount to total protein amount in the sample, determined by absorbance measurements at 420 nm and 750 nm, respectively, using the Beckman DU-68 Spectrophotometer, respectively. The total protein level was assayed by the Lowry method (BIO-RAD). The experiments were done with n = 3–4 replicates.

### Fluorometry

Cells were gently rinsed with PBS and 200 μl/well of PBS was added. Measurement of fluorescence was performed using a Wallac Victor 3 microplate fluorometer at excitation and emission wavelengths of 485 nm and 535 nm, respectively. Results were presented as fluorescence ratio of mean values of three measurements from three to six independent experiments versus average readout of control experiments—non-treated cells or cells treated with: AP-15/DOPE/DMEM:pSec, AP-15/DOPE:pSec, AP-15/DOPE, AP-15/DOPE/DMEM, DOPE and AP-15 (autofluorescence).

### Western blot analysis

The TIMP2 protein amount was determined in the growth medium collected from cell cultures as well as in the cell lysates and tissues. Cell lysates were prepared using RIPA buffer with the addition of Protease Inhibitor Cocktail (Sigma-Aldrich) according to the protocol from Abcam. Pellets from cells were suspended in ice cold buffer, incubated for 20 min at 4°C and centrifuged at 12 500 rpm for 20 min at 4°C. The supernatants were collected and assayed for total protein concentration using the Lowry method (Bio-Rad). Protein extraction from melanoma tissues was performed according to the protocol described by Haq et al. [23], with minor changes. The melanoma tissue (100mg) from each mouse was individually pulverised in liquid nitrogen, suspended in 0.1 M Tris buffer (pH 8.1) containing protease inhibitors, sonicated for 10 cycles (30 sec sonication, 30 sec pause) at 4°C and ultracentrifuged (100 000 g, 45 min, 4°C). The collected supernatants as well as the media collected from cell cultures were centrifuged in Amicon Ultra-4 Centrifugal Filter Unit columns (Merck Millipore) according to the manufacturer’s protocol. The dense fraction was collected and assayed for total protein concentration using the BCA Protein Assay (Pierce) or the Bradford method (Bio-Rad). Western blots were carried out according to standard procedures with an SDS-PAGE Electrophoresis System (Bio-Rad). Primary monoclonal anti-TIMP2 antibody (Cell Signaling #5738 TIMP2 Rabbit mAb), 1:1000 dilution, secondary antibody conjugated to HRP (sc-2030, Santa Cruz) and secondary antibody conjugated to AP (#170–6515, Bio-Rad) were used. Analysis with monoclonal anti-GAPDH primary antibody, (MAB374, Merck Millipore), 1:500 dilution, AP (#170–6515, Bio-Rad) conjugated secondary antibody and HRP conjugated secondary antibody (sc-2031, Santa Cruz) was performed as a positive control. Detection was performed with the enhanced chemiluminescence method (ECL TM Western blotting detection reagents and analysis system) or with a colorimetric detection system (NBT-BCIP solution, Sigma).

### Crystal Violet assay

Cells were washed with PBS and 0.5% crystal violet solution was added to each well. After 10 min of incubation the plate was washed three times with water and dried at room temperature overnight. Fixed dye was dissolved in 33% acetic acid solution and measured spectrophotometrically using a plate reader (Perkin Elmer Victor 3 Model 1420–012) at a wavelength of 540 and 690 nm.

Cell viability was calculated as the percentage of control cells. Experiments were performed in triplicate.

### Microscopic image analysis

Photographs were taken using a Nikon DS-U2-Fi1c camera attached to Nikon’s Eclipse Ti inverted microscope.

### RT-PCR and qPCR

Total RNA was isolated using the TRI Reagent (Ambion, The RNA Company). Single-stranded cDNA was synthesized using the High Capacity cDNA Reverse Transcription Kit (Life Technologies). Expression of lipid regulated genes was examined on TaqMan Array 96-Well Plate Mouse Lipid Regulated Genes (Life Technologies) according to the manufacturer’s instructions. Transcript level of target genes was normalized to the mRNA level of housekeeping genes encoding: hypoxanthine phosphoribosyltransferase 1 (*Hprt1*) and β-glucuronidase (*Gusb*) and calculated using the DDCt method. Experiments were performed in duplicate.

### *In vivo* gene transfer experiments

Animal studies were performed in accordance with the procedures in Department of Genetics in Maria Sklodowska-Curie Memorial Cancer Center and Institute of Oncology, with permission of the Bioethics Committee of the Medical University of Warsaw (Permit Numbers: 27/04/08 and 48/2012). C57BL/6 and BALB/c mice of both sexes, aged 4–6 weeks, were used. Animals were housed at a constant temperature of 21°C with a 12-hour light/12-hour dark cycle. All procedures were carried out under general anaesthesia with inhaled isoflurane (Forane, Abbot Japan, Tokyo, Japan), and all efforts were made to minimize suffering.

### Gene transfer to B16-F10 tumor-bearing C57BL/6 mice

Tumors in C57BL/6 mice were induced by subcutaneous injection of 2–3 × 10^5^ B16-F10 cells in 100 μl PBS. 11–13 days after tumor cell inoculation the mice were divided into seven groups and gene formulations were administered intratumorally as two doses injected with a one hour break (2x100 μl). The following experimental groups were analyzed: 1) H_2_O (n = 3), 2) AP-15 (2 x 14.5 μg of lipid) (n = 3), 3) AP-15/DOPE (2 x 5 μg of lipids) (n = 3), 4) pSec (2 x 100 μg) (n = 3), 5) pTIMP2 (2 x 100 μg) (n = 3), 6) AP-15:pTIMP2 complexes (n = 4) or 7) AP-15/DOPE:pTIMP2 complexes (n = 4), injected twice, containing pTIMP2 (2 μg) and 14.5 μg or 5 μg of lipids, respectively. 3 days post injection the mice were sacrificed and the tumors were harvested.

Genomic DNA from B16-F10 tumor isolated with Genomic Mini (A & A Biotechnology) according to the protocol provided by the manufacturer, was subjected to PCR amplification with primers complementary to the coding sequence of the *TIMP2* gene: F: 5’AAGAATTCCTGCAGCTGCTCCCCGG3’, R: 5’AACTCGAGCCACAGGGGCGTTGGAG3’. PCR products were separated on a 1.5% agarose gel (Sigma).

### Gene transfer to L1 tumor-bearing BALB/c mice

Ascites tumors in BALB/c mice were induced by intraperitoneal injection of 2.5 × 10^5^ L1 cells in 100 μl PBS. One week after tumor cell inoculation the gene formulations were administered intraperitoneally as a single injection (800 μl). The mice, divided into five groups, received: 1) H_2_O (n = 2), 2) AP-15 (181 μg of lipids) (n = 2), 3) AP-15/DOPE/DMEM (62.5 μg of lipids) (n = 2), 4) AP-15:pTIMP2 complexes (n = 7) or 5) AP-15/DOPE/DMEM:pTIMP2 complexes (n = 7), comprised of pTIMP2 (25 μg) and 181 μg or 62.5 μg of lipids, respectively. 3 days post injection the mice were sacrificed and exudates were collected.

Lysis of erythrocytes was performed using hypotonic lysis buffer (0.15 M KHCO_3_, 1 mM NH_4_Cl, 0.1 mM Na_2_EDTA, H_2_O). First, 5 volumes of erythrocyte lysis buffer was added to exudates, samples were incubated on ice for 10 min with occasional stirring, and afterwards centrifuged at 3000 rpm for 15 minutes at 4°C. The resulting pellet was suspended in 2 volumes of lysis buffer and lysis of erythrocytes was repeated 4–5 times until a pure L1 sarcoma cell pellet was obtained.

Genomic DNA from L1 cell pellet was isolated and subjected to PCR analysis as described above.

### Statistical analysis

Quantitative data were obtained using the Shapiro-Wilk normality test. In case of data for which deviations from the normal distribution had not been shown (p> 0.05 in the Shapiro-Wilk test) differences between the compared samples were assessed using Student's t-test. The data not complying the condition of normal distribution was analysed by the Mann—Whitney U test or the Kruskal-Wallis test. The threshold of significance was set at 0.05.

## Results

### AP:pDNA complex formation

**Fig 2:**
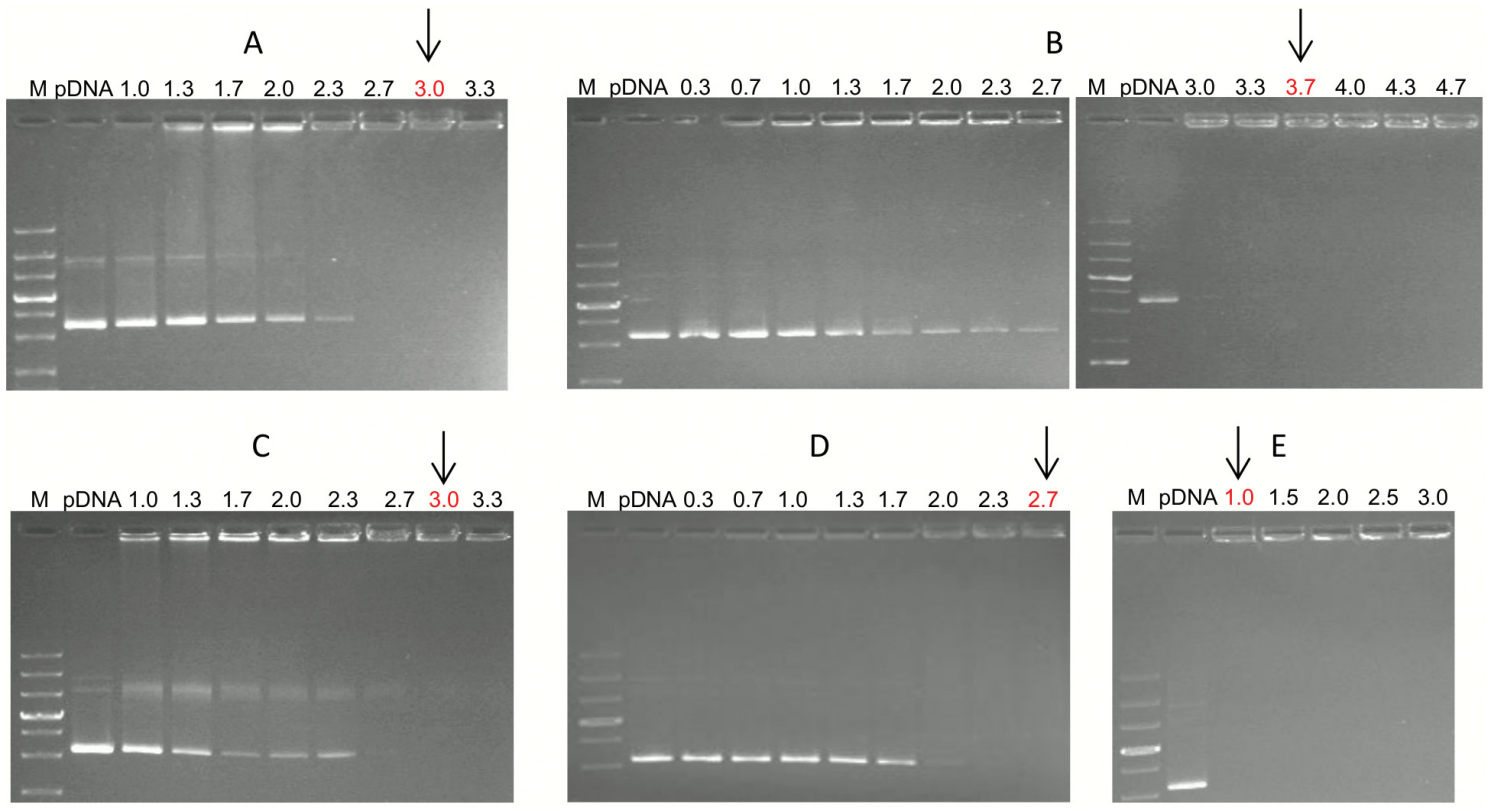
Gel retardation analysis with different carrier:pDNA complexes at various N/P ratios (of amino-group of the carriers to the phosphate group of the nucleic acid). (a) AP-11:pDNA, (b) AP-15:pDNA, (c) AP-7:pDNA, (d) AP-8:pDNA, (e) PEI:pDNA. M- molecular weight size marker 1kb+.

AP were studied for DNA binding ability using electrophoresis retardation assay at the various AP to pDNA ratios from 0.3 to 4.0 as expressed as N/P (Fig 2). DNA migration in agarose gels is retarded, when the carrier:pDNA complex is formed. Moreover, when the entire amount of DNA is bound in the complex its migration is entirely abolished [24]. The complex formation was observed in the gel at approximate N/P ratio r ≥ 2.7 for AP-8, r ≥ 3.0 for AP-7 and AP-11, r ≥ 3.7 for AP-15, whereas for PEI:DNA complex migration was retarded below ratio r ≤ 1.0.

Total complexation of pDNA occurred when 22.5μg of lipids/μg of pDNA was used, similar results were obtained for both types of AP-15 and DOPE-containing complexes (see S5 Fig for AP-15/DOPE:pDNA complexes).

### Transfection efficiency *in vitro*

#### Transfection using AP:pGFP complexes

**Fig 3:**
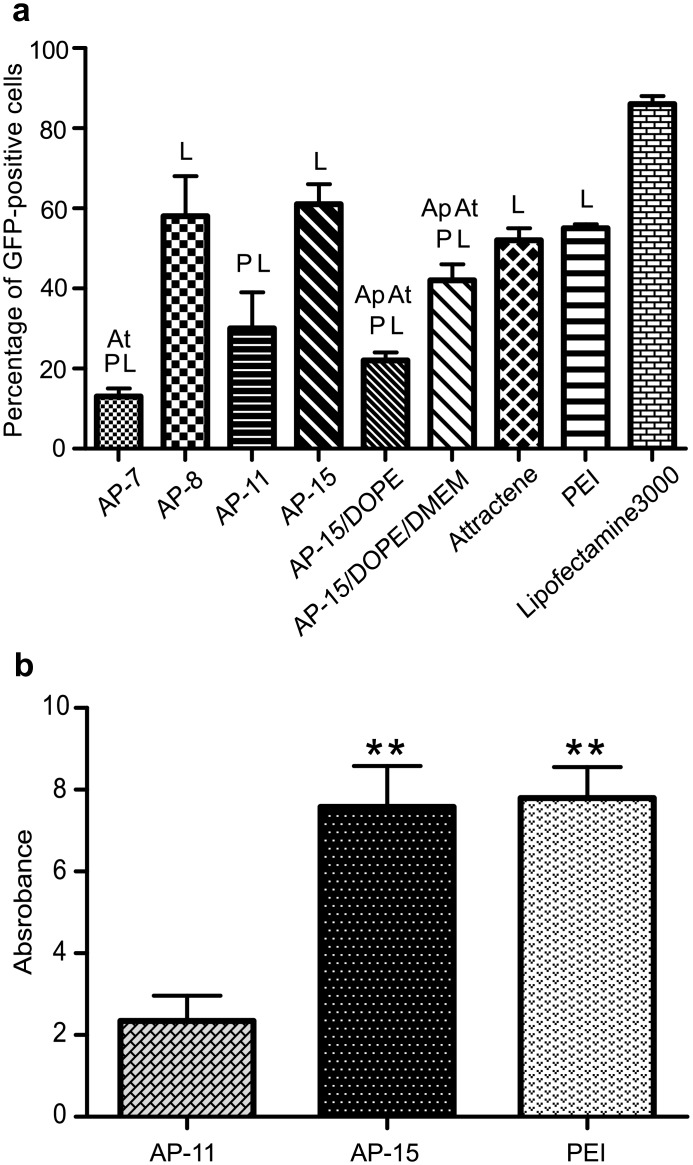
Evaluation of transfection efficiency of B16-F10 cells transfected with use of carrier:pDNA complexes. (a) Percentage of GFP-positive cells transfected with complexes: AP:pGFP at different N/P ratios (r = 1.7 for AP-7, AP-8, AP-11, r = 2.0 for AP-15) and AP-15/DOPE:pGFP, AP-15/DOPE/DMEM:pGFP lipoplexes containing 2.5 μg of lipids/μg of pGFP, analysed by FACS; Ap- significant difference from AP-15 treatment, At- significant difference from Attractene treatment, L- significant difference from Lipofectamine treatment, P- significant difference from PEI treatment, (b) Activity of β-galactosidase in cells transfected with complexes: AP-11:pLacZ, AP-15:pLacZ, PEI:pLacZ, at r = 2.0–2.5 N/P ratio, studied by β-Gal test; *P<0.05, **P<0.005. Transfections were performed using 4 μg of respective pDNA.

The effect of AP on efficacy of transfection was assessed using a pDNA bearing an GFP encoding gene (Fig 3a), in this experiment 4 μg of pGFP was used per each transfection. Before subjecting to flow cytometry analysis, the cells transfected with the formulations containing optimal amounts of pDNA and respective APs in the most appropriate N/P ratio (what resulted in introducing pDNA to the cells with the highest efficiency) were selected using microscopic observations. Flow cytometry analysis of transfected B16-F10 cells confirmed high transfection efficiency for complexes containing AP-8 and AP-15. The highest numbers of GFP-positive cells—58% and 61% of viable GFP expressing cells were noticed for complexes with AP-8 (r = 1.7) and AP-15 (r = 2.0), respectively. These numbers were higher than those recorded for commercial agents—Attractene (52% of viable GFP-positive cells), PEI (55% of viable GFP-positive cells), however lower than estimated for Lipofectamine3000 (86% of viable GFP-positive cells). The complexes containing AP-7 (r = 1.7) and AP-11 (r = 1.7) transfected the cells with lower efficiency (approx. 13% and 30% of viable GFP-positive cells, respectively) (Fig 3a).

#### Transfection using AP:pLacZ compexes

To further confirm the results obtained with use of GFP-encoding plasmid transfection of B16-F10 cells with AP:pLacZ complexes followed by colorimetric estimation of nitrophenol was performed. High β-galactosidase activity was demonstrated in cells transfected with complexes AP-15:pLacZ (Fig 3b). This activity was comparable with that obtained with PEI:pLacZ complexes at similar doses (4 μg pLacZ, r = 2–2.5), and higher than the activity observed in cells transfected with AP-11:pLacZ (Fig 3b). Interestingly, APs transfect the cells more effectively in N/P ratios lower than optimal for complex formation, approximately estimated in an agarose gel retardation assay. Transfection with complexes containing AP-11 and AP-15 was around 2-fold more effective at ratios 1.7 and 2.0, respectively, than that noted at higher N/P ratios, irrespectively of pDNA amounts (S1 Fig and data not shown).

#### Transfection using AP-15/DOPE/DMEM:pGFP, AP-15/DOPE:pGFP and AP-15/DOPE:pTIMP2 complexes

In order to optimize the transfection efficacy, two types of formulations containing AP-15 and helper lipid DOPE were prepared. The amounts of lipids optimal for transfection was estimated. Similar results were obtained for AP-15/DOPE/DMEM:pDNA and AP-15/DOPE:pDNA complexes, namely the best transfection efficiency was achieved when 2.5 μg of lipids/1 μg of pDNA was used (data for AP-15/DOPE/DMEM are shown in S6 Fig).

**Fig 4:**
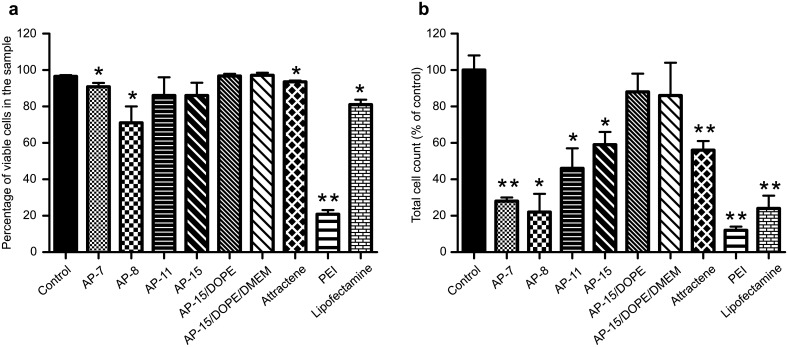
(a) cell viability and (b) total cell number of B16-F10 cells transfected with AP:pGFP complexes at various N/P ratios (r = 1.7 for AP-7, AP-8, AP-11, r = 2.0 for AP-15), or with AP-15/DOPE:pGFP and AP-15/DOPE/DMEM:pGFP lipoplexes at 2.5 μg of lipids/μg of pDNA dose, analysed by FACS. Transfections were performed with the use of 4 μg of pDNA. *P<0.05, **P<0.001.

Furthermore, microscopic analysis revealed that transfection efficiency of AP-15/DOPE:pGFP complexes was substantially higher than the efficiency of Lipofectamine3000 (S2a and S2c Fig). Surprisingly, the results of flow cytometry indicated that Lipofectamine3000 transfected the cells more efficiently than AP-15/DOPE/DMEM or AP-15/DOPE transfection mixtures (Fig 3a). It should be kept in mind, however, that percentage of GFP-positive cells is dependent on the total number of the cells in the sample. In case of Lipofectamine3000 transfected cells the total cell count was considerably lower than that of cells transfected with AP-15 and DOPE-containing lipoplexes as indicated by flow cytometry (Fig 4b). Thus, our results suggest that Lipofectamine3000 significantly inhibited cell proliferation.

Flow cytometry analysis showed also that AP-15 and DOPE-containing lipoplexes transfected the cells with efficiency lower than AP-15:pGFP complexes. However the transfectional efficacy of AP-15/DOPE/DMEM:pGFP complexes (42% of GFP-positive cells) was only slightly lower than those of commercial agents Attractene and PEI (52% and 55% of GFP-positive cells, respectively) (Fig 3a). This might imply that DOPE-containing formulations require further optimization and it will be a subject of future studies.

**Fig 5:**
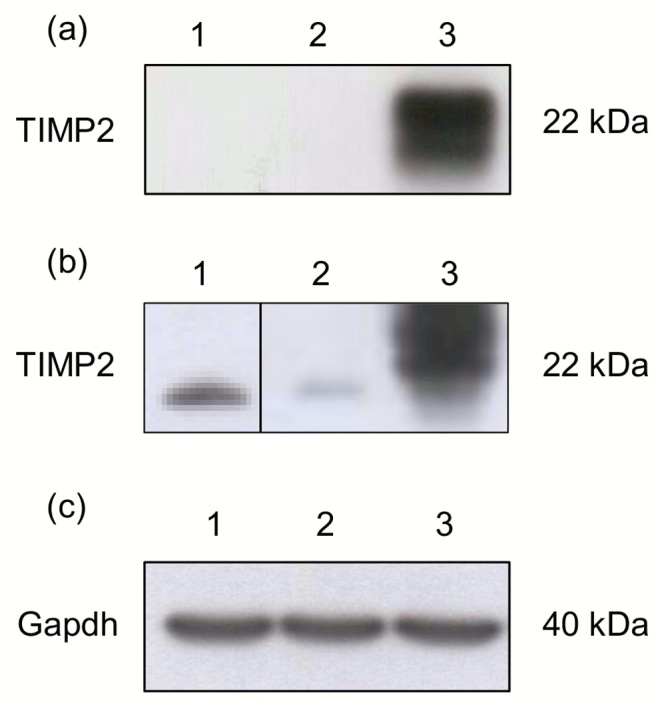
TIMP2 protein expression in B16-F10 cells assessed by Western blot in. (a) cell lysates, (b) media from cells. (c) Control Gapdh protein level in cell lysates. Lines: 1- B16-F10 cells, 2- B16-F10 cells transfected with AP-15/DOPE:pGFP, 3- B16-F10 cells transfected with AP-15/DOPE:pTIMP2.

It was further shown that AP-15/DOPE transfection mixture efficiently introduced the therapeutic *TIMP2* gene into B16-F10 cells, where it was effectively expressed. The B16-F10 cells transfected with AP-15/DOPE:pTIMP2 produced higher amounts of TIMP2 protein than control cells (Figs 5 and S4).

### Cell number, viability and morphology of cells transfected with APs

#### Cells transfected with AP:pGFP complexes

**Fig 6:**
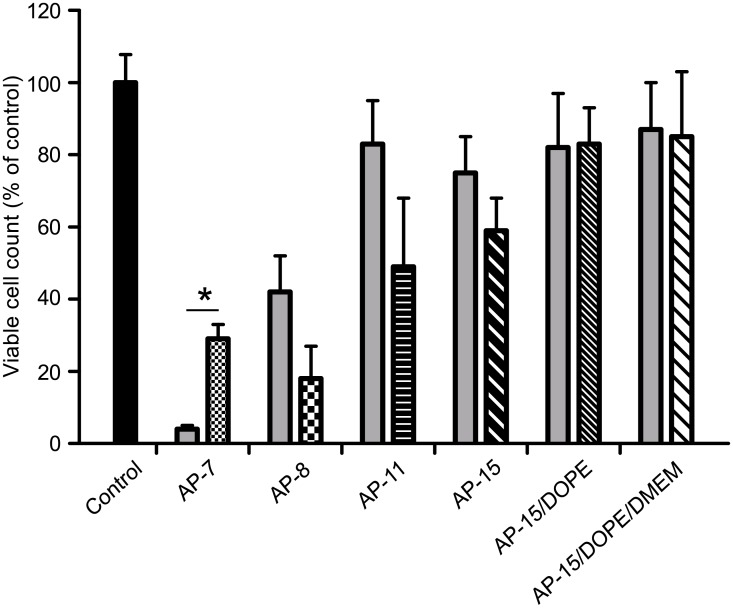
Number of viable cells in samples treated with the various APs / AP-based reagents alone (grey bars: AP-7, AP-8, AP-11, AP-15, AP-15/DOPE, AP-15/DOPE/DMEM) and AP:pGFP / AP-15 and DOPE-containing complexes (patterned bars: AP:pGFP: AP-7, AP-8, AP-11 r = 1.7, AP-15 r = 2.0; AP-15/DOPE:pGFP, AP-15/DOPE/DMEM:pGFP 2.5 μg of lipids/μg of pDNA), analysed by FACS. Transfections were performed with the use of 4 μg of pDNA. *P<0.05.

7-AAD-staining of non-viable cells allowed their subsequent discrimination with use of flow cytometry from viable cells in the sample. Study with 7-AAD showed a high viability of cells treated with AP:pGFP complexes (71–91%, Fig 4a). However, in the preliminary studies, in the case of cells transfected with AP-7 and AP-8 the viability and the number of cells studied under the microscope was very low. Therefore, in contrast to low-toxic AP-11 and AP-15, during the transfection procedure with AP-7 and AP-8 the media containing the latter APs had to be replaced by fresh standard (APs-free) media. The viability and number of cells transfected with AP-8 complexes containing 2 or 3 μg pGFP was higher than that observed for complexes with 4 μg of pDNA, whereas the transfection efficiency was similar (data not shown). Interestingly, the number of the cells in the samples subjected to transfection with APs, shown as percentage of non-transfected cells, was decreased for cells transfected by complexes with AP-8 and-7 (total cell count 28% and 23% of the control, respectively) (Fig 4b). Such an effect on cell number was not observed when complexes containing AP-15 or AP-11 were used (total cell count was approximately 59% and 46% of the control, respectively; Fig 4b). Effects of cell treatment with free APs alone, apart from AP-7, resulted in smaller decreases of cell viability (decrease in viable cell count by 96%, 58%, 17% and 25% for AP-7, -8, -11 and -15, respectively) than that observed for AP:pGFP complexes (Fig 6). It should be emphasized that AP-15:pGFP complex triggered lower decrease in cell number than Atractene, PEI and Lipofectamine (total cell count 56%, 12% and 24% of the control, respectively) (Fig 4b).

#### Cells transfected with AP-15/DOPE/DMEM:pGFP or AP-15/DOPE:pGFP complexes

AP-15/DOPE/DMEM:pGFP complexes caused rather low decrease in B16-F10 cell viability percentage for all the doses tested (to 80%), whereas Attractene affected the viability of cells more significantly (percentage reduction to 18%) (S7 Fig).

The crystal violet assay, based on the study of metabolic capacity of the cells, did not allow to differentiate the cytotoxic effect from proliferation inhibition, cytostatic effect and possible cell death. Therefore, on the basis of observations in inverted (data not shown) and confocal microscope with Hofmann contrast of B16-F10 cells transfected with AP-15/DOPE:pGFP complexes the cells did not exhibit any characteristic morphological changes indicative for necrosis or apoptosis. In contrast, in samples transfected with Lipofectamine3000 the presence of many constricted cells was observed (S2b and S2d Fig).

Flow cytometry analysis showed that AP-15/DOPE/DMEM:pGFP and AP-15/DOPE:pGFP complexes did not influence either the cell viability or the total cell count of transfected cells (97% of viable cells for both types of complexes, 86% and 88% of total cell count, respectively) (Fig 4a and 4b). Those numbers were considerably higher than that estimated for commercial transfection reagents- Attractene, PEI and Lipofectamine (Fig 4). Moreover, transfecting formulations did not affect the number of viable cells either when applied on cells alone or when complexed with pDNA (88% and 85% of viable cell count for AP-15/DOPE/DMEM, 86% and 84% of viable cell count for AP-15/DOPE, respectively) (Fig 6).

### Expression of lipid metabolism-related genes in cells transfected with AP-15/DOPE:pDNA complexes

The transfection using various carriers may affect the expression of different off-target genes, involved in diverse cellular pathways. To verify this hypothesis we screened transcript levels of 44 genes related to lipid metabolism in B16-F10 cells transfected with AP-15/DOPE:pSec or AP-15/DOPE:pTIMP2 complexes using a TaqMan^®^ Array Mouse Lipid Regulated Genes (Table 1). In cells transfected with AP-15/DOPE:pSec an increased mRNA level of six genes (*Abca1*, *Adfp*, *Cd36*, *Nr1h3*, *Slc27a1*, *Tnf*) with fold change ranging from 1.66 to 3.71 and a reduction of the transcript level of one gene (*Soat 2*, fold change 0.33) was observed. In transfected cells gene transcript of: *Abcg1*, *Alox5*, *Alox15*, *Cyp27a1*, *Fabp4*, *Il1b*, *Lpl*, *Ltc4s*, *Ptgs2*, *Tbxas* genes was not detected. The level of gene expression for: *Alox5ap*, *Fabp5*, *Il6*, *Lta4h*, *Ppard*, *Pparg* i *Ucp2* genes could not be estimated accurately.

**Table 1:** Effect of transfection of B16-F10 cells with AP-15/DOPE:pDNA complexes on expression of lipid metabolism-related gene.

Gene	B16-F10	B16-F10/AP-15/DOPE:pSec(fold change relative to control ± SD)	B16-F10/AP-15/DOPE:pTIMP2(fold change relative to control ± SD)	Involvement in cellular process
***Abca1***	1 (0.56–1.8)	**2.43 (1.41–4.19)**	**2.1 (1.45–3.06)**	Reduction of cellular level of cholesterol
***Acadvl***	1 (0.56–1.8)	1.38 (0.8–2.38)	1.35 (0.94–1.93)	Catalysis of the initial step of the mitochondrial fatty acid beta-oxidation pathway
***Acat1***	1 (0.54–1.85)	0.72 (0.4–1.28)	0.81 (0.54–1.21)	Catalysis of to the synthesis of cholesteryl esters (using long chain fatty acyl coenzyme A) for intracellular storage
***Adfp***	1 (0.56–1.79)	**1.66 (0.96–2.85)**	**1.98 (1.38–2.85)**	Possible marker of lipid accumulation in cells
***Alox12***	1 (0.52–1.91)	0.6 (0.34–1.05)	0.35 (0.24–0.53)	Catalysis of oxidation of polyunsaturated fatty acids with molecular oxygen
***Apoe***	1 (0.56–1.79)	1.2 (0.7–2.07)	1.15 (0.8–1.66)	Intracellular transport and metabolism of lipids
***Cd36***	1 (0.53–1.9)	**2.65 (1.54–4.56)**	1.1 (0.63–1.91)	Membrane transport of long-chain fatty acids (LCFA)
***Fads1***	1 (0.55–1.83)	0.99 (0.56–1.77)	1.03 (0.7–1.51)	Fatty acid desaturase (formation of PUFA)
***Fads2***	1 (0.55–1.81)	1.18 (0.68–2.06)	1.08 (0.75–1.56)	- ǁ -
***Fads3***	1 (0.55–1.83)	0.7 (0.39–1.27)	0.73 (0.49–1.09)	No function confirmed
***Gyk***	1 (0.54–1.85)	0.94 (0.54–1.64)	0.93 (0.63–1.39)	Glycerol uptake and metabolism
***Hadhb***	1 (0.52–1.91)	1.11 (0.61–2.03)	**1.67 (1.16–2.4)**	β-subunit of mitochondrial trifunctional protein—involved in mitochondrial fatty acid β-oxidation pathway
***Hmgcr***	1 (0.55–1.81)	1.09 (0.62–1.93)	1 (0.69–1.47)	Biosynthesis of precursors for cholesterol and isoprenoids
***Hmgcs1***	1 (0.54–1.86)	0.97 (0.53–1.77)	0.94 (0.64–1.36)	Biosynthesis of precursors for cholesterol and isoprenoids
***Insig1***	1 (0.55–1.82)	0.84 (0.48–1.47)	0.67 (0.46–0.98)	Regulator of HMGCR
***Ldlr***	1 (0.56–1.8)	1.06 (0.61–1.82)	1.06 (0.72–1.56)	Cholesterol uptake
***Nr1h3***	1 (0.55–1.83)	**1.66 (0.95–2.89)**	**3.02 (2.1–4.36)**	Reduction of cellular level of cholesterol
***Pla2g4a***	1 (0.53–1.88)	1.3 (0.75–2.25)	1.25 (0.79–1.99)	Arachidonic acid formation (hydrolysis of phospholipids); implicated in signal transduction, apoptosis, inflammation
***Scd1***	1 (0.56–1.8)	1.27 (0.74–2.19)	1.01 (0.7–1.45)	Catalysis of the conversion of saturated fatty acids to monounsaturated fatty acids; likely to have wide-ranging effects on cellular membrane physiology, energy storage, and signaling
***Slc16a6***	1 (0.56–1.8)	0.82 (0.47–1.41)	0.67 (0.47–0.96)	Monocarboxylate Transporters family member with no attributed function
***Slc27a1***	1 (0.56–1.8)	**1.85 (1.07–3.21)**	**2.08 (1.44–3.01)**	Transport of LCFA across biological membranes, cholesterol metabolism regulation
***Slc27a3***	1 (0.55–1.81)	1.11 (0.64–1.9)	1.31 (0.9–1.9)	Catalysis of fatty acid activation but not fatty acid transport
***Soat2***	1 (0.55–1.81)	**0.33 (0.19–0.58)**	0.78 (0.53–1.14)	Esterification of cholesterol in the endoplasmic reticulum; Inhibition of *Soat2* expression in liver cells in mice resulted in lower total cholesterol in their serum.
***Srebf1***	1 (0.55–1.81)	1.22 (0.68–2.18)	1.12 (0.75–1.66)	Regulation of the expression of genes encoding enzymes required for fatty acid and cholesterol biosynthesis
***Srebf2***	1 (0.55–1.83)	1.14 (0.66–1.98)	1.1 (0.76–1.6)	- ǁ -
***Stard4***	1 (0.54–1.84)	1.17 (0.68–2.03)	1.07 (0.74–1.54)	Postulated role in intracellular sterol transport or/and cholesterol biosynthesis
***Tnf***	1 (0.54–1.85)	**3.71 (2.09–6.57)**	**3.65 (2.5–5.31)**	Inhibition of the uptake of free fatty acids and promotion of lipogenesis, induction of lipolysis, inhibition of the activity of lipid metabolism enzymes

### *In vivo* transfection efficiency

**Fig 7:**
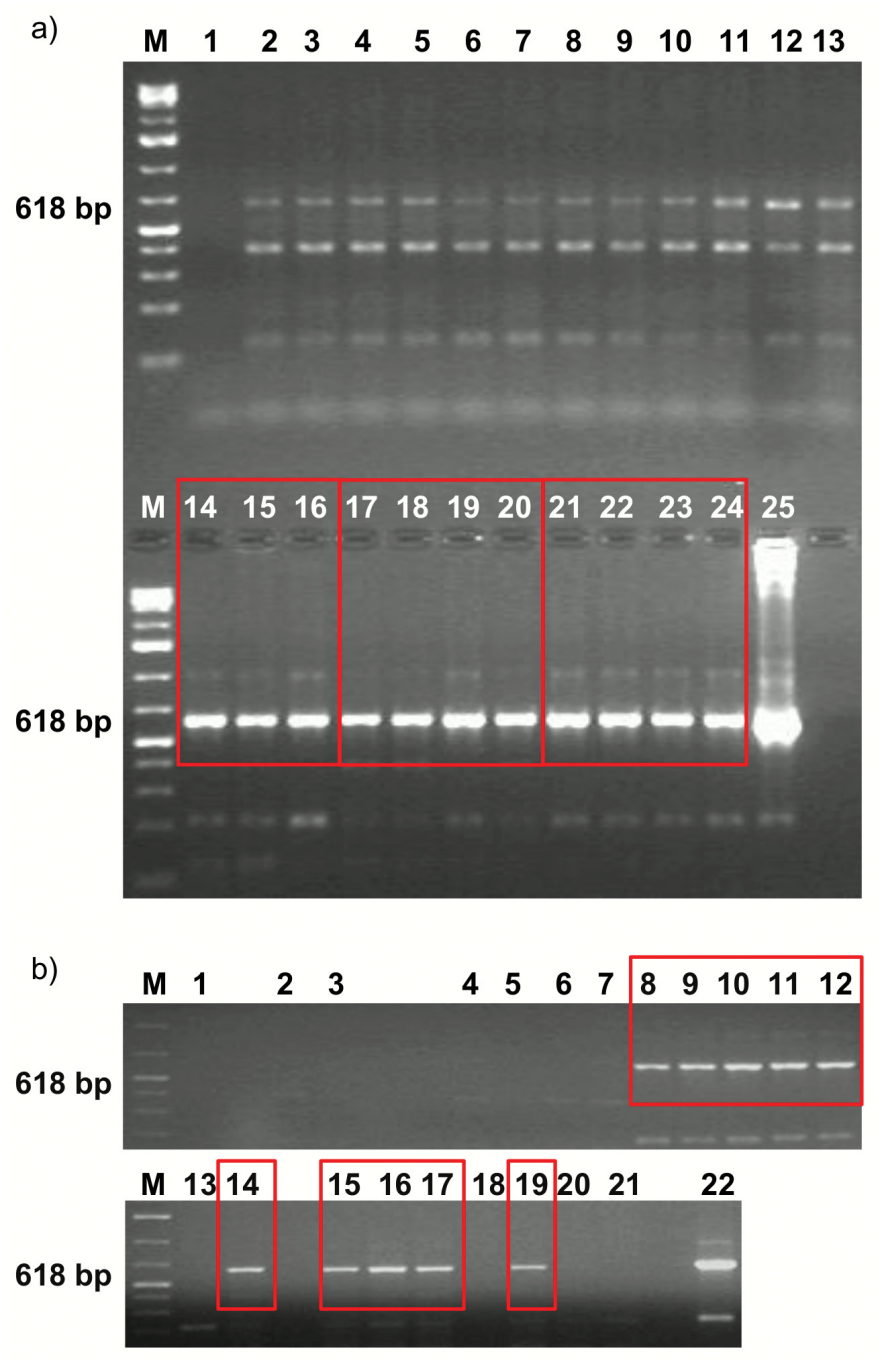
*In vivo* transfection study of tumor cells using AP-15-based complexes. Analyses of PCR products using DNA templates from: (a) B16-F10 tumors, injected with: 2-4- H_2_O, 5-7- AP-15/DOPE, 8-10- AP-15, 11-13- pSec, 14-16- pTIMP2, 17-20- AP-15/DOPE:pTIMP2 complexes, 21-24- AP-15:pTIMP2 complexes; 25- positive control for PCR (pTIMP2), (b) L1 tumors, injected with: 2-3- H_2_O, 4-5- AP-15, 6-7- AP-15/DOPE/DMEM, 8-14- AP-15:pTIMP2 complexes, 15-21- AP-15/DOPE/DMEM:pTIMP2 complexes; 25- positive control for PCR (pTIMP2). M- molecular weight size marker 1kb+, 1- reagent control for PCR.

To determine whether complexes introduce therapeutic genes *in vivo* we injected gene formulations systemically and topically to mice bearing L1 and B16-F10 tumors. AP-15:pTIMP2 and AP-15/DOPE:pTIMP2 complexes transfected the B16-F10 tumors with high efficacy (4/4 samples positive), comparable to that obtained with use of the sole pTIMP2 plasmid applied in a 50-fold greater dose (Fig 7a). We also found that AP-15:pTIMP2 complexes efficiently transfected L1 tumors (6/7 samples positive), whereas the efficiency of AP-15/DOPE/DMEM:pTIMP2 complexes was lower (4/7 samples positive), but it seems that still adequate, considering the route of specimen administration (Fig 7b).

However, in B16-F10 tumor cells transfected with AP-15:pTIMP2 and AP-15/DOPE:pTIMP2 complexes the enhanced level of TIMP2 protein was not observed indicating that the expression of the therapeutic gene in tumor cells was not efficient or the expressed protein rapidly degraded (S3 Fig).

## Discussion

In this study we examined the use of semi-synthetic cationic derivatives of polyprenols, Amino-Prenols (AP), as components of transfecting mixtures.

### Formation of AP:pDNA complexes

First we studied whether these substances possess DNA binding ability. AP:pDNA complexes were efficiently formed, most probably due to electrostatic interactions between the positively charged quaternary AP salts and the negatively charged DNA molecules. It is generally believed that efficiency of retardation of the electrophoretic mobility of complexed pDNA is positively correlated with the stability of thus formed complexes [25]. However, better DNA complexation ability of cationic carriers has not always been associated with higher efficiency of genetic material delivery to cells as shown by Kearns et al. [26] and by Goyal et al. [27]. Flow cytometry data described here indicate that a complex built of AP-15, for which complete complexation of pDNA occurs at r≥3.7 N/P ratio, introduces pDNA into cells with a very high efficiency (61%) at the ratio r = 2.0. Complexes containing AP-11, for which the optimum of complex formation was r≥3.0, transfected cells with moderate efficiency (approx. 30%) at r = 1.7 (Fig 3a). These results are consistent with previous reports showing that the charge ratio of cationic lipid:DNA required for complete complexation of DNA was different for various cationic lipids, such as DMRIE, DOSPA, N,N-dioctadecyllysineamide, N^1^,N^1^-dioctadecyl-1,2,6-triaminohexane, DOTAP, but was overall lower than in that observed for APs [28, 29]. Therefore, results of the retardation assay should be carefully considered and should not be discussed as a decisive marker of transfection efficacy of a particular cationic lipid. Additionally, analysis of the binding of AP-15/DOPE to pDNA by using the gel retardation assay revealed that, comparing to AP-15, complexation efficiency of AP-15/DOPE is decreased (S5 Fig). Literature data on the effect of DOPE on complexation of pDNA with cationic lipids are limited. Misra et al. [30], by means of EtBr exclusion assay, showed that inclusion of DOPE to cationic cholesterol lipids resulted in the reduction of their DNA binding efficiency. The effect of DOPE on APs binding ability to DNA requires systematic studies to be performed in the future. Still, it should be kept in mind that complete complexation of DNA is not an essential condition of efficient transfection as discussed above.

For lipids with saturated alkyl chains (C18-C14 N-[1-(2,3-dioleyloxy)propyl]-N,N,N-trimethlylammonium chloride (DOTMA) or spermine derivatives) the reduction of the carbon chain length was found to be associated with improved transfection efficiency [31, 24]. However, this does not seem to be the general rule. Depending on the type of the substance tested, higher transfection efficiency was obtained with lipids with shorter or longer alkyl chain lengths [32, 33]. For AP lipids studied in this report, the viability and cell count of transfected cells suggest their divergent response to AP prenyl chain length. Variable and chain-length dependent effects of various APs on cell viability and proliferation are not easy to explain and require further studies. This most probably results from the different physico-chemical interactions of APs with cellular membrane lipids. Moreover, the metabolic turnover of various APs might differ.

Results of transfection experiments of various APs should be carefully elucidated keeping mind that the toxicity of the AP-7 and -8 was considerably higher than that of longer-chain APs. For this reason the media containing AP-7 or AP-8 had to be removed after 5 h and replaced by standard serum-supplemented growth medium for the subsequent 43 h in order to analyze the effects of transfection. Such procedure is recommended by manufacturers of several commercial toxic transfection agents. In the case of non-toxic AP-11 or -15 cells were incubated for 48 h without removing the media. It seems that the long hydrophobic chain of AP-15, together with the cationic group of this polyprenyl derivative, allows the formation of stable complexes with pDNA, ensuring physico-chemical properties adequate for cell transfection. This might probably explain why AP-15:pDNA complexes possess a high capacity to transfect cells. According to Adhikari et al. [34] any amphipathic molecule with hydrophilic/lipophilic balance is able to make aggregates in aqueous solutions. For example aggregates called “micelles” (with hydrophobic core) can be formed in aqueous solution by ionic molecule conjugated with a single lipophilic carbon chain. This molecule can form “reverse micelle” (with hydrophilic pocket in the inside core) in organic solvent [34]. DNA and cationic lipids mixed in solution form aggregates spontaneously [35]. Cationic lipids bind pDNA by electrostatic interactions between positively charged groups of lipids and negatively charged phosphates of pDNA backbone, or by formation of hydrogen bonds between the positively charged amino groups and hydroxyl groups of cationic lipids and pDNA [36]. It is also described in the literature that transfection efficiency depends on the biophysical characteristics of the aggregates, rather than on the chemical structure of lipid. On the basis of study of Massing et al. [37] on analogs of DOTAP (1,2-dioleoyl-3-(trimethyammonium) propane) with various carbon chain length it was shown that the transfection efficiency of resulting aggregates (lipoplexes) depends on their biophysical properties [38]. Shi et al. [39] demonstrated in their studies on carbamate-linked cationic lipids with different hydrocarbon chains that lipid containing long unsaturated chain (C18:1) have better transfection efficiency than lipid with saturated carbon chain (C18:0). The authors suggest that increased length of the saturated alkyl chain decreases the fluidity of the bilayer which can lead to potential disruption of the endosome and enhanced possibility of DNA degradation. They describe, however, that lipid with long unsaturated chain (e.g. C18:1) can be easily fused with membranes due to their high fluidity and this promotes DNA release, leading to higher transfection efficiency and lower cytotoxicity [39]. Also Scarzello et al. [40] who tested the physicochemical and transfectional properties of Sunfish cationic amphiphiles showed, that the presence of longer carbon tails or unsaturation of the lipids appeared to be beneficial for transfection efficiency. Taking these literature data into account cationic derivatives of polyisoprenoid alcohols, containing highly unsaturated hydrocarbon chains, were applied for cell transfection despite the fact that the mechanism of self-aggregation of APs remains elusive. It might be also speculated that the increase of the polyisoprenoid chain-length results in its increased hydrophobicidy and might induce stronger intramolecular hydrophobic interactions. Consequently, the longer polyisoprenoid chain is, more stable aggregates are formed. This highly speculative supposition awaits, however, experimental verification. Also, we have demonstrated that the efficiency of transfection depends on the N/P ratio. We found that for each polyprenyl derivative the optimal amount of pDNA for transfection of a given number of cells may be different, while the best (+/-) charge ratio of complexes is similar. For all the carriers tested the transfection occurred most effectively at the values of (N/P) ratios of complexes lower than those, which were determined by means of a gel retardation assay (S1 Fig and data not shown). On the one hand this might suggest that, in the case of APs, complete complexation of plasmid is not required for highly efficient gene transfer. Similar observations were made by Sun et al. [33] who found that the highest transfection efficiency was exhibited by cells transfected with complexes formed at that N/P ratio for which 80% but not 100% of pDNA binding was shown. Additionally, Saito et al. [41] demonstrated that in order to obtain the best transfection efficiency optimal charge ratio for galactosylated complexes was r = 2.3 and transfection efficiency was reduced at higher charge ratios. Such an effect may be due to the cytotoxicity of the cationic carrier, whose concentration is higher at higher r [33]. Still, the r values determined for lipofecting agents should be interpreted with caution. Such numbers indicate the possibility of interaction between the molecules of cationic lipid and nucleic acid, and might suggest the possible steichiometry of the complex formed. Moreover, the absolute r values should not be considered as a discriminating criterion for rating of the lipofecting potential of compounds of interest. We used the charge ratio values obtained in the retardation assay to obtain a preliminary estimate of the lipid to DNA ratio in the complexes, rather than to make conclusive statements on the ability of APs to bind DNA.

### Efficiency of transfection with AP-based complexes

Lipofectamine2000 or PEI are considered in the literature as a gold standard for lipid or polymer-mediated gene transfection [27, 42]. Numerous reports aimed at construction of novel lipid vectors concluded that the newly studied formulas did not ensure satisfactory results compared to these standards. In this report we demonstrated that APs are able to efficiently introduce different plasmids into cells at a level comparable to that achieved with commercially available transfection reagents.

Additionally, Mochizuki et al. [43] synthesized a cationic lipid comprising ethylene diamine (DA) which showed very low if any transfection efficiency. Moreover, transfection with the commonly used cationic lipid DOTAP appeared to be very inefficient. In order to obtain higher transfection efficiency, in both cases the above-mentioned helper lipid DOPE was used, which greatly improved the obtained results [43]. Also others showed that addition of DOPE to novel cationic carriers resulted in substantial enhancement of their transfection activity, which was then comparable to or higher than the transfection activity of commercial lipofecting agents [32, 33, 44]. The role of DOPE in such transfecting formulas is still under investigation. On the one hand it is postulated that cationic lipids are responsible for the destabilization of the endosomal membrane since they induce a conformational change of negatively charged membranes [45, 46]. On the other hand, there are indications that DOPE can stimulate the formation of an inverted non-lamellar (hexagonal) structure of complexes, facilitating fusion between the liposomal and endosomal bilayers and subsequent plasmid DNA release. This implies a higher transfection efficiency—DNA may be released from the endosomal compartment with higher efficiency, moved to the nucleus and subjected to transcription [31, 47]. However, it was mentioned in the literature that cationic lipids able to form lamellar phase do not inevitably undergo structural phase transition to hexagonal structure upon DOPE inclusion in those bilayers [48]. Nevertheless, high affinity of polyisoprenonoids towards phosphatidylethanolamine observed in model membranes [13] gave rise to the concept of considering DOPE as a co-lipid for transfection.

It should be emphasized that solely AP-7, without addition of DOPE, transfected the DU145 cells with very low efficiency, whereas supplementation of this phospholipid to the transfection formulation substantially improved the transfection effectiveness, and appeared comparable to that obtained with Lipofectamine2000 [10]. In the light of the above data, two types of transfection formulations, containing AP-15 and helper lipid DOPE were tested in this report. It was found that AP-15/DOPE/DMEM:pGFP and AP-15/DOPE:pGFP complexes were less efficient than AP-15:pGFP complexes, however the transfection efficacy of AP-15/DOPE/DMEM:pGFP was only slightly lower than that of commercial agents Attractene and PEI. It should be remembered, however, that the total cell count in samples transfected with the DOPE containing formulations was considerably higher than in those transfected with the commercial agents (see also section Toxicity of APs). Moreover, Rosenzweig et al. [49] showed that addition of DOPE to diquaternary ammonium salts, which as pure lipids transfected the cells with efficiency comparable to commercially available reagents such as Lipofectin, Lipofectace or Lipofectamine, resulted in the reduction of their transfection efficacy. Additionally, inclusion of DOPE did not improve transfection efficiency of complexes containing lipospermine [50]. In turn Bouxsein et al. [51] showed that presence of DOPE in complexes containing DOTAP and siRNA resulted in nonspecific silencing effect, increased toxicity and did not improve silencing efficiency of DOTAP:siRNA complexes. Similarly, DOPE inclusion did not improve transfection efficiency of cationic lipids in *in vivo* systems [52–54]. All together these reports might indicate that DOPE is not an “universal enhancer” of transfection. Further studies are required to clarify the effect of DOPE on tranfsection potential of APs containing complexes.

Carriers of genetic material are aimed at delivery of the genes encoding therapeutic proteins into the cells of the multicellular organism. Moreover, the tested gene should be expressed at an appropriate level. We demonstrated, in the *in vivo* experimental model, that complexes containing AP-15 and pTIMP2 administered to mice bearing B16-F10 or L1 tumors introduced the *TIMP2* gene to tumor cells with high efficiency (Fig 7a and 7b). However, likely because of the low dose of the plasmid in the formulations, the introduced gene was probably not or insignificantly expressed in C57BL/6 mice, as shown at the protein level (S3 Fig).

Numerous non-viral carriers of genetic material effectively transfected cells at relatively high pDNA doses and sometimes after repeated administration. Louis et al. [55] showed, by means of the PCR method, that PEI:pDNA complexes introduced the plasmid into target tissue of mice after intraperitoneal administration with satisfactory efficacy only when 100 μg of pDNA per injection was applied. It was also shown [56], that DOTMA/Chol:pCMV-Luc complexes, after intravitreal injection to rabbits, resulted in significantly higher luciferase activity observed in the vitreous body than that obtained with naked pDNA alone when 85 μg of pCMV-Luc was used [56]. Probably, complexes with AP-15 described in this report, administered either intraperitoneally or intratumorally to mice, contained too low amount of plasmid (in total as little as 25μg or 4μg of pTIMP2 plasmid, respectively) to obtain high level of transgene expression.

Moreover the anti-TIMP2 antibody which worked perfectly on the growth medium collected from cell cultures could be not sensitive enough to detect TIMP2 protein from tissue by Western blot method. It is worth to underline that TIMP2 is the protein which is mainly secreted to the extracellular matrix, easy to obtain in *in vitro* culture, whereas hard to extract from tissues. Also, *in vivo*, cells are under the influence of constant paracrine and exocrine signalling required for tissue differentiation and maintenance [57], what could have influence on TIMP2 concentration. TIMP2, in contrary to the housekeeping genes, which are very stable and easy to detect, is probably unstable. It has a half-life of only 4.5 minutes [58]. In an organism there are many levels on which determination of steady state protein concentration occurs, such as transcription, transcript degradation, regulation of post-transcription, translation and protein degradation, which cannot be undervalued in protein expression level evaluation studies [59]. Despite the fact that Lichtinghagen et al. [60] observed higher MMP-9 protein levels in cancer tissue than in normal tissues obtained from 17 patients with prostate cancer they did not show the significant correlation between the mRNA and protein expression of MMP-2, MMP-9 and TIMP-1 in either cancerous or noncancerous tissue. All these reasons should be taken into account, when considering why we did not obtain high level of TIMP2 protein expression in B16-F10 melanoma tumors.

### Studies on toxicity of APs

Application of new efficient transfection carriers for introducing genetic material into living organisms absolutely requires their low cytotoxicity [61]. The tested AP:pDNA complexes did not considerably affect the percentage of viable cells in the samples (Fig 4a). However, when the results were expressed as percent of the control (non-treated cells) free APs and AP:pDNA complexes caused a decrease in viable B16-F10 cell counts (Fig 6). Our results show that the APs derivatives, except for AP7, in complexes with pDNA influenced the number of viable cells more profoundly than those lipids applied on cells alone. Interestingly, such effect was not observed for AP-15/DOPE complexes. The effect of AP:pDNA complexes might be related to the interactions between cationic carrier and pDNA. In line with this Nguyen LT et al. [62] demonstrated that widely used cationic lipid reagent Lipofectamine2000 did not significantly affect the viability of B16BL6 melanoma cells, rat glioma cells and human cervical carcinoma cells. However, the use of complexes of this reagent with pDNA resulted in significant toxicity toward these cells, which the effect increased with the carrier content in the complexes [62]. Nonetheless, AP-15:pDNA complexes had a lower inhibitory effect on cell proliferation than the commercial lipid transfection reagents Attractene, Lipofectamine3000 or PEI (Fig 4b), similarly to other transfectionally effective, non-toxic derivatives of cationic lipids or polymers, reported in the literature [27, 42].

The effect of DOPE on the reduction of cationic carrier cytotoxicity towards cells was previously described in the literature [43, 44]. We have shown that the AP-15/DOPE:pGFP and AP-15/DOPE/DMEM:pGFP complexes at the dose optimal for transfection caused very small decrease in cell viability (S7 Fig) as well as in total and viable cell counts. Worth noting is the protective effect of DOPE, used as the component of the transfection mixture, on cell proliferation. Moreover, Gawrys et al. [15, 16] showed in *in vivo* experiments, that AP-7/DOPE liposomes did not affect, after subcutaneous administration, the overall condition, behaviour nor the function of the cardiovascular and renal excretory system of rats. Though in our cell-line studies AP-7 influenced B16-F10 cell proliferation, it seems that this compound and hopefully other Amino-Prenols too, are non-toxic for living organisms and can be used in various applications.

### APs influence on metabolism of cell lipids

It was shown [63], that transfection with use of different types of vectors changes the expression of the off-target genes. Bentinger et al. [64] demonstrated that all-*trans* epoxidized polyisoprenoid lipids influenced the lipid metabolism of cells by inducing CoQ and simultaneously inhibiting cholesterol biosynthesis in HepG2 human hepatoblastoma cells. However, the effect of epoxidized poly-*cis* polyisoprenoids on the synthesis of these compounds is negligible [64]. To verify the effect of APs on lipid metabolism-related genes we analysed the expression of selected appropriate genes in cells transfected with complexes containing AP-15 by means of real-time PCR. Our analysis showed that the expression level of six and one gene, out of 44 genes tested, was increased or reduced, respectively—all these genes were involved in the regulation of cholesterol homeostasis (Table 1). Further studies are required, however, to clarify the effect of APs on cholesterol metabolism.

## Conclusions

In this study we demonstrated that Amino-Prenols, semi-synthetic derivatives of polyprenols, may serve as non-viral carriers for gene delivery. We found that all the APs tested (AP-7, AP-8, AP-11 and AP-15) have DNA complexation ability. We further showed that transfection occurred most effectively at the values of (N/P) ratios of AP:pDNA complexes lower than those, which were estimated as theoretically optimal for transfection. This suggests that complete complexation of plasmid with AP is not a prerequisite for high efficiency of gene transfer. Additionally, we found that among the APs tested, AP-15 most efficiently introduced pGFP or pLacZ to cells. It should be emphasized that transfection efficiency of AP-15 was higher than that obtained for the commercial reagent Attractene or polymer PEI. It is important to underline that AP-15 had a lower inhibitory effect on cell number than Attractene or PEI and the viability of transfected cells was high. We also showed that AP-15 and DOPE-containing complexes deliver pDNA into the cells with efficiency only a little lower than Attractene or PEI and influence the cell number to a considerably lesser extent than the commercial agents. However, we found that efficiency of *TIMP2* therapeutic gene delivery into cells *in vivo* by AP-15 was high for the low dose of pTIMP2 plasmid applied in both types of formulations, we did not observe the high expression of introduced transgene at the protein level. In summary, AP-15 has a high potential as a carrier of genetic material for *in vitro* applications.

## Supporting Information

S1 Fig Activity of β-galactosidase in cells, studied by β-Gal test. Cells were transfected with AP-11:pLacZ or AP-15:pLacZ complexes containing 8 μg or 6 μg pLacZ, respectively, at various N/P ratios, **P<0.005.
(EPS)

S2 Fig Morphology of cells transfected with (a) AP-15/DOPE:pGFP (2 μg pGFP, 5.0 μg of lipids), viewed under confocal fluorescence microscope; (b) AP-15/DOPE:pGFP (2 μg pGFP, 5.0 μg of lipids), viewed under Hoffman modulation contrast in a confocal microscope; (c) Lipofectamine3000:pGFP viewed under a confocal fluorescence microscope; (d) Lipofectamine3000:pGFP, viewed under Hoffman modulation contrast in a confocal microscope. (TIF)

S3 Fig The comparison of protein level of (a) Gapdh, (b) TIMP2 in B16-F10 tumors injected with: 1- AP-15, 2- AP-15/DOPE, 3- pSec, 4,5- pTIMP2; 6,7- AP-15:pTIMP2; 8,9- AP-15/DOPE:pTIMP2, M- protein ladder 10–250 kD. The membrane was cut in two parts, the upper part was incubated with anti-Gapdh antibody, whereas lower part was incubated with anti-TIMP2 antibody.
(TIF)

S4 Fig Protein expression in B16-F10 cells assessed by Western blot. TIMP-2 protein level in: a) cell lysates and c) media from cells, b) Gapdh protein level in cell lysates.
(TIF)

S5 Fig Gel retardation analysis with AP-15/DOPE:pDNA complexes at various μg of lipids/μg of pDNA ratios. M- molecular weight size marker 10kb.
(TIF)

S6 Fig Efficacy of transfection of B16-F10 cells with AP-15/DOPE/DMEM:pGFP complexes containing different amounts of lipids studied by fluorometric measurement. The transfection performed with 2 μg of pGFP. *P<0.05.
(EPS)

S7 Fig Viability of cells transfected with AP-15/DOPE/DMEM:pGFP complexes at different amounts of lipids, studied by crystal violet test. The transfection performed with 2 μg of pGFP. **P<0.005.
(EPS)
